# Development of a PCR based marker system for easy identification and classification of aerobic endospore forming bacilli

**DOI:** 10.1186/2193-1801-2-596

**Published:** 2013-11-09

**Authors:** Sangeeta Kadyan, Manju Panghal, Khushboo Singh, Jaya Parkash Yadav

**Affiliations:** Department of Genetics, M. D. University, Rohtak, 124001 Haryana India

**Keywords:** AEFB, 16S rRNA gene, HaeIII, Specificity of fragment, PCR, *In silico*

## Abstract

Restriction fragment length analysis of 16S rRNA gene of 52 different aerobic endospore forming Bacilli (AEFB) strains with HaeIII enzyme has revealed the presence of a 460 bp long fragment in 50 AEFB strains. BLAST analysis revealed that the fragment was 463 bp long and it was located at 3’ end of 16S rRNA gene. Further specificity of this fragment for AEFB strains was checked by PCR and *in silico* methods. In PCR based method a primer pair (463 F and 463R) specific to this fragment was designed and this primer pair has shown amplification of 463 bp fragment in AEFB strains only. In *in silico* methods homology of primer pair and presence of restriction enzyme site in 16S rRNA genes were checked in 268 species of AEFB. Almost all species of AEFB have shown positive results for both of the tests. Further multiple alignments of 463 bp sequences of different species of AEFB have shown that it is a good marker for identification and classification of AEFB.

**Electronic supplementary material:**

The online version of this article (doi:10.1186/2193-1801-2-596) contains supplementary material, which is available to authorized users.

## Introduction

Aerobic endospore-formers have long been considered to be important components of the soil bacterial community (Mandic-Mulec and Prosser 2011). There is a great diversity of physiology among the aerobic spore formers. Their collective features include degradation of all substrates derived from plant and animal sources including cellulose, starch, pectin, proteins, agar, hydrocarbons and others, antibiotic production, nitrification, denitrification, nitrogen fixation, facultative lithotrophy, autotrophy, acidophily, alakliphily, psychrophily, thermophily and parasitism. Endospore formation, universally found in this group, is thought to be a strategy for survival even under adverse soil environment, where these bacteria predominate (Kumar et al. 2012). To get the beneficial effects of these AEFB it becomes very necessary to know how much diverse and abundant these microbes are in different soil ecosystems. Since 1990s various approaches based on phenotypic and genotypic characteristics have been applied to identify and classify the members of class Bacilli. Few decades before genus *Bacillus* was the only representative of class Bacilli among aerobic spore formers. Development of cultivation independent approaches have attracted microbiologist towards the molecular approaches for examining the microbes in a better way. Among different molecular methods, 16S rRNA gene sequencing is the best one. Since 1991, several new genera of aerobic spore formres like *Amphibacillus* (Niimura et al. 1990), *Paenibacillus* (Ash et al. 1991, 1993), *Alicyclobacillus* (Wisotzkey et al. 1992), *Aneurinibacillus* (Shida et al. 1996), *Brevibacillus* (Shida et al. 1996), *Gracilibacillus* (Waino et al. 1999), *Salibacillus* (Waino et al. 1999), *Virgibacillus* (Heyndrickx et al. 1998), *Filobacillus* (Schlesner et al. 2001), *Geobacillus* (Nazina et al. 2001), *Jeotgalibacillus* and *Marinibacillus* (Yoon et al. 2001) and *Ureibacillus* (Fortina et al. 2001) have been created based on this method. For phylogenetic arrangement of these newly discovered texa various markers based on 16S rDNA have been developed by different scientists (Priest et al. 1988; Ash et al. 1991; Gurtler and Stanisich 1996; Daffonchio et al. 1998a, b; Goto et al. 2000; Stackebrandt and Swiderski 2002; Xu and Cote 2003; De Clerck et al. 2004; Vardhan et al. 2011). Primer set developed by Garbeva et al. (2003) was found to be 100% specific for many of species of *Bacillus* and related genera. After a gap of years, Vardhan et al. (2011) developed a set of primers for identification of hyper variable region of 16S rDNA in different *Bacillus* species and partial sequencing of this hyper variable region behaves as an index for easy identification of species related to genera *Bacillus*.

With development of more advanced approaches to find cultivable and noncultivable diversity of microbes, lot of new species and genera, belonging to AEFB are discovering day by day. So, need of new marker systems is always there for proper identification and classification of these lineages. Hence the main objective of present study was to develop a simple and easy identification and classification tool for *Bacillus* and related genera which is an extension of research related to bacilli. The restriction digestion of amplified 16S rRNA gene by HaeIII enzyme has given a fragment of around 460 bp length in all species of *Bacillus* and related genera. Sequence information of this fragment (downloaded from NCBI) was used to find exact length of the fragment (463 bp) and to develop specific primers for amplification of this fragment in AEFB genera. Further sequence information and multiple alignment of 463 bp long sequences of different species of AEFB genera has revealed that this is an easy tool for identification and classification of the members of *Bacillus* and related genera. Another beneficial information provided by our study is that almost all species of *Bacillus* and related genera have restriction enzyme sites for Hae III enzyme which give a product of 460 bp. Restriction enzyme site for HaeIII are present at different positions in other bacterial lineages, therefore give product of different size after restriction digestion which clearly discriminate the *Bacillus* and related genera from others.

## Material and methods

### Bacterial strains

All of the bacterial strains used in the present study are Bacilli isolated from the rhizospheric soil of *Phyllanthus amarus* which were identified by 16S rRNA gene sequencing in our previous research work (Kadyan et al. 2013). Taxonomic information and accession numbers of isolates have been given in Table 1.

**Table 1:** **Strain names and NCBI accession numbers of 52 AEFB strains isolated from rhizospheric soil of** ***Phyllanthus amarus***

Strain code	Bacterial isolate	Accession number	Strain code	Bacterial isolate	Accession number
1.P3	*B. marisflavi* JP44SK40	JX129227	15.P2	*B. subtilis* subsp. *spizizenii* JP44SK24	JX144714
2.P1	*B. megaterium* JP44SK1	JX144691	16.P1	*B. simplex* JP44SK25	JX144715
2.P2	*B. megaterium* JP44SK2	JX144692	16.P2	*B. simplex* JP44SK26	JX144716
3.P1	*Lysinibacillus sphaericus* JP44SK3	JX144693	17.P3	*B. cereus* JP44SK27	JX144717
3.P2	*Lysinibacillus sphaericus* JP44SK4	JX144694	18.P3	*B. aquimaris* JP44SK28	JX144718
3.P3	*B. megaterium* JP44SK5	JX144695	19.P1	*B. simplex* JP44SK29	JX144719
4.P1	*B. licheniformis* JP44SK6	JX144696	19.P2	*B. simplex* JP44SK30	JX144720
5.P3	*Paenibacillus taiwanensis* JP44SK7	JX144697	20.P1	*B. simplex* JP44SK31	JX144721
6.P1	*B. mycoides* JP44SK8	JX144698	20.P2	*B. simplex* JP44SK32	JX144722
6.P3	*B. mycoides* JP44SK9	JX144699	23.P1	*B. cereus* JP44SK33	JX144723
7.P1	*B. aryabhattai* JP44SK11	JX144701	23.P2	*B. cereus* JP44SK34	JX144724
7.P2	*B. megaterium* JP44SK10	JX144700	23.P3	*B. megaterium* JP44SK35	JX144725
7.P3	*Lysinibacillus xylanilyticu* s JP44SK52	JX155769	24.P1	*B. mycoides* JP44SK36	JX144726
8.P1	*B. simplex* JP44SK12	JX144702	24.P3	*B. cereus* JP44SK37	JX144727
8.P2	*B. simplex* JP44SK13	JX144703	25.P2	*B. aryabhattai* JP44SK38	JX144728
8.P3	*B. arsenicus* JP44SK14	JX144704	26.P3	*B. megaterium* JP44SK39	JX144729
9.P3	*B. marisflavi* JP44SK15	JX144705	27.P1	*Brevibacillus laterosporus* JP44SK41	JX155758
10.P3	*B. firmus* JP44SK16	JX144706	27.P3	*B. cereus* JP44SK42	JX155759
11.P1	*B. firmus* JP44SK17	JX144707	30.P1	*B. cereus* JP44SK43	JX155760
11.P3	*B. megaterium* JP44SK18	JX144708	31.P3	*Jeotgalibacillus* sp. JP44SK56	KC012993
12.P3	*B. flexus* JP44SK19	JX144709	36.P3	*B. cereus* JP44SK44	JX155761
13.P1	*B. megaterium* strain JP44SK21	JX144711	37.P3	*B. cereus* JP44SK45	JX155762
13.P3	*B. firmus* JP44SK20	JX144710	38.P3	*Terribacillus saccharophilus* JP44SK46	JX155763
14.P2	*Brevibacillus laterosporus* JP44SK51	JX155768	41.P3	*Terribacillus goriensis* JP44SK47	JX155764
14.P3	*B. cereus* JP44SK22	JX144712	43.P3	*B. cereus* JP44SK49	JX155766
15.P1	*B. subtilis* subsp. *spizizenii* JP44SK23	JX144713	44.P3	*B. mycoides* JP44SK50	JX155767

### 16S rRNA gene amplification and restriction digestion by HaeIII enzyme

**Figure 1:**
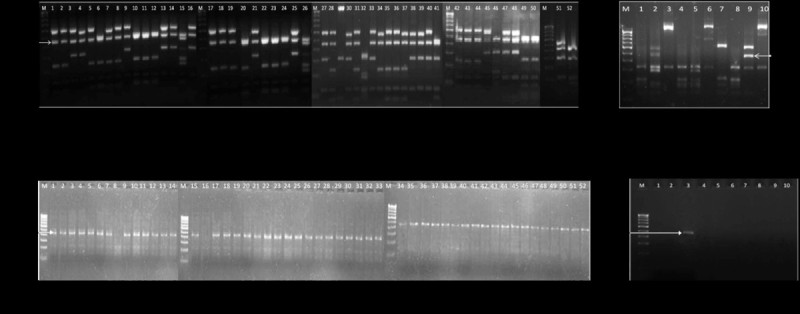
**Gel photograph showing ARDRA pattern of 52 AEFB strains (a) Gel photograph of ARDRA pattern of 52 AEFB strains digested with Hae III restriction enzyme.(b)** Gel photograph of ARDRA pattern of 10 reference strains digested with Hae III restriction enzyme. **(c)** Gel photograph of PCR amplified 463 bp fragments in 52 strains of AEFB. **(d)** Gel photograph of PCR amplification result of 463 bp fragments in 10 reference strains. **(a)** Lane M - 100 bp DNA marker. Lanes 1–52 indicate bacterial strain codes (2.P1, 3.P1, 4.P1, 6.P1, 7.P1, 8.P1, 11.P1, 13.P1, 15.P1, 16.P1, 19.P1, 20.P1, 23.P1, 24.P1, 27.P1, 30.P1, M, 2.P2, 3.P2, 7.P2, 8.P2, 15.P2, 16.P2, 19.P2, 20.P2, 23.P2, 14.P2, M, 1.P3, 3.P3, 5.P3, 6.P3, 7.P3, 8.P3, 9.P3, 10.P3, 11.P3, 12.P3, 13.P3, 14.P3, 17.P3, 18.P3, 24.P3, M, 23.P3, 25.p2, 26.P3, 27.P3, 31.P3, 36.P3, 37.P3, 38.P3, 41.P3, 43.p3, 44.p3). **(b)** Lane M -100 bp DNA marker, lane 1–10 *Shigella flexneri* ATCC12022, *Proteus mirabilus* ATCC43071, *Staphylococcus aureus* ATCC259323, *E. Coli* ATCC25922, *Salmonella typhimurium* ATCC13311, *Klebsiella pneumoniae* ATCC 700603, *Pseudomonas fluorescens* MTCC1749, *Serretia marrcescens* MTCC4822, *Bacillus subtilis* MTCC7193, *Staphylococcus aureus* MTCC7443. Arrow indicates the size of 460 bp fragment in *Bacillus subtilis* MTCC7193. **(c)**: M - 100 bp DNA marker, lane 1–52 (1.P3, 2.P1, 2.P2, 3.P1, 3.P2, 3.P3, 4.P1, 5.P3, 6.P1, 6.P3, 7.P1, 7.P2, 7.P3, 8.P1, 8.P2, 8.P3, 9.P3, 10.P3, 11.P1, 11.P3, 12.P3, 13.P1, 13.P3, 14.P2, 14.P3, 15.P1, 15.P2, 16.P1, 16.P2, 17.P3, 18.P3, 19.P1, 19.P2, 20.P1, 20.P2, 23.P1, 23.P2, 23.P3, 24.P1, 24.P3, 25.P2, 26.P3, 27.P1, 27.P3, 30.P1, 31.P3, 36.P3, 37.P3, 38.P3, 41.P3, 43.P3, 44.P3). Arrow indicates the size of fragment. **(d)**: Bacterial strain *Bacillus subtilis* MTCC7193, present in lane no. 3 has shown amplification of 463bp fragment and other reference strains have not shown any amplification. Arrow indicates the size of fragment compared with marker of 100 bp present in lane M.

Gene coding for 16S rRNA gene of all of the 52 AEFB strains along with 10 reference strains (*Shigella Flexneri* ATCC12022*, Proteus mirabilus* ATCC43071*, Staphylococcus aureus* ATCC259323*, E. Coli* ATCC25922, *Salmonella typhimurium* ATCC13311, *Klebsiella pneumonia* ATCC 700603*, Pseudomonas fluorescens* MTCC1749, *Serretia marcescens* MTCC4822, *Bacillus subtilis* MTCC7193, and *Staphylococcus aureus* MTCC7443) was amplified by using universal primers i.e. B27f (5'-AGAGTTTGATCCTGGCTCAG-3') and U1492R (5'- GGTTACCTTGTTACGACTT-3') in thermal cycler (Biorad). Further reaction mixture for restriction digestion was prepared by mixing 8.5 μl of purified PCR products, 5 U of restriction endonuclease, HaeIII (Fermentas) and 1.0 μl of 10X recommendation buffer. Reaction mixture was incubated overnight in water bath at 37°C. Restriction digested DNA was analysed by horizontal electrophoresis in 2% agarose gels with 100 bp DNA marker. The gels were visualized on a gel documentation system (Alpha Innotech). Photograph of gel has been shown in Figure 1(a&b).

### Restriction pattern analysis and designing of oligonucleotide primers

Restriction pattern analysis of HaeIII digested 16S rRNA gene has shown the presence of a fragment having length around 460 bp (Figure 1a) in all of the bacterial species belonging to *Bacillus* and related genera (except *Bacillus arsenicus*, *Paenibacillus taiwanensis* and 9 reference strains related to other bacterial lineages) (Figure 1b). On the basis of these observations it was assumed that this 460 bp fragment was specific for *Bacillus* and related genera. To find out the exact location and sequence information of this fragment, 16S rRNA gene sequence of all of the *Bacillus* isolates taken in our study was downloaded from NCBI gene bank database. All of the 16S rRNA gene sequences were checked for HaeIII enzyme cut sites (GG↓CC). Sequence between two cut sites having length of around 460 bp was found in all of the 16S rRNA gene sequences at same position. Further length of this region was found to be 461-463 bp. Primer pair specific to this region was designed by using software, Primer 3.0 and further synthesized from the facility available at Eurofins Genomics India Pvt. Ltd., Bangalore.

### Sequence specificity of primer pair and occurrence of restriction enzyme site

The specificity of oligonucleotide primers was checked by PCR amplification of the 463 bp fragment in all of the 52 AEFB isolates along with 10 reference strains (*Shigella Flexneri* ATCC12022*, Proteus mirabilus* ATCC43071*, Staphylococcus aureus* ATCC259323*, E. Coli* ATCC25922, *Salmonella typhimurium* ATCC13311, *Klebsiella pneumonia* ATCC 700603*, Pseudomonas fluorescens* MTCC1749, *Serretia marcescens* MTCC4822, *Bacillus subtilis* MTCC7193, and *Staphylococcus aureus* MTCC7443). Reaction conditions for PCR were, initial denaturation at 94°C for 5 minutes, 30 cycles of denaturation at 95°C for 30 seconds, annealing at 55°C for 20 seconds, extension at 72°C for 30 seconds and at last final extension at 72°C for 7 minutes. Theoretically primer pair was checked for its specificity in 16S rRNA gene sequences (downloaded from NCBI) in different species of *Bacillus* and related genera i.e. 153 different species of *Bacillus*, 20 *Virgibacillus,* 15 *Geobacillus,* 1 *Filobacillus,* 4 *Jeotgalibacillus,* 5 *Ureibacillus,* 21 *Alicyclobacillus*, 5 *Amphibacillus*, 5 *Aneurinibacillus*, 16 *Brevibacillus*, 9 *Gracilibacillus*, 5 *Paenibacillus*, 5 *Lysinibacillus* and 4 *Terribacillus*. A number of other bacterial lineages of Gram positive and negative bacteria were also checked for primer specificity which includes genera from phylum Firmicutes (other than Bacilli), Actinobacteria, Alpha Proteobacteria, Beta Proteobacteria and Gamma Proteobacteria.

### Multiple alignment of 463 bp long partial 16S rDNA sequence of different species of Bacilli

To check the ability of marker for classification of Bacilli, we have done the multiple alignments of specific, 463 bp long sequences of 16S rRNA gene of 52 strains (taken in our study) with the reference sequences downloaded from NCBI. Multiple alignment of very closely related species of genus *Bacillus* (29 different species of *Bacillus*) lying in two nearby clusters in all species living tree by Yarza et al. (2010) has also been done to check the differentiation ability of this sequence. Software Clustal X 2.0 (Larkin et al. 2007) was used for alignment of different sequences and further alignment file was used in molecular evolutionary genetic analysis software version 5.1 (MEGA 5.1) (Tamura et al. 2011) for construction of phylogenetic tree.

## Results

### Oligonucleotide primers

*Bacillus* and related genera specific primers designed in our study were named as 463 F (5’CTAAAACTCAAAGGAATTGACG3’) and 463R (5’AATACGTTCCCGGGCCTT3’).

### PCR amplification of 463 bp sequence

PCR amplification has confirmed the specificity of the primer pair in 52 AEFB strains and 10 reference strains. Out of total, 50 strains belonging to *Bacillus* and related genera have shown the amplification of the specific region. However, the region was not amplified in *Bacillus arsenicus*, *Paenibacillus taiwanensis* and 9 reference strains (Figure 1c & d).

### Sequence homology of primers in 16S rRNA gene sequences of ***Bacillus*** and related genera

Primer sequences were found to be 100% similar with the 16S rRNA gene sequences (downloaded from NCBI) of 120 species of genera *Bacillus*, 13 *Geobacillus,* 1 *Filobacillus,* 4 *Jeotgalibacillus,* 5 *Ureibacillus,* 7 *Alicyclobacillus*, 2 *Brevibacillus* and 5 *Lysinibacillus*. Number of other bacterial lineages of Gram positive and negative bacteria which includes genera from phyla Firmicutes (*Staphylococcus chromogenes* D83360, *Streptococcus pyogenes* AB002521, *Enterococcus faecalis* AB012212, *Clostridium populeti* X71853, *Listeria monocytogenes X56153*), Actinobacteria (*Corynebacterium diphtheria* X84248, *Mycobacterium tuberculosis* X58890, *Nocardia asteroids* AF430019, *Streptomyces lavendulae* subsp. *Lavendulae* D85116), Alpha proteobacteria (*Rhizobium leguminosarum* U29386, *Azospirillum lipoferum* Z29619, *Acetobacterium woodii* X96954), Beta proteobacteria (*Burkholderia cepacia* U96927, *Bordetella pertussis* U04950) and Gamma Proteobacteria (*Pseudomonas aeruginosa* X06684, *Escherichia coli* X80725, *Klebsiella pneumoniae* X87276, *Shigella dysenteriae* X96966) have not shown any sequence homology (Table 2).

**Table 2:** **% similarity of 463 bp sequence of 16S rRNA gene of type sp. (** ***Bacillus subtilis*** **) with 16S rRNA sequences of different AEFB strains (downloaded from NCBI), primer sequences in these AEFB strains, presence and absence of restriction enzyme site and position of specific fragment in AEFB strains**

Sr. no.	Name of bacteria	NCBI accession no.	Sequence of primer pair in different AEFB strains	% similarity of 463 bp sequence and presence of restriction enzyme site	Position of 463 bp sequence in 16S rRNA gene
1	*Alicyclobacillus sacchari*	AB264020	AAT**CC** GTTCCCGGGCCTT CTGAAACTCAAAGGAATTGACG	88%+	913-1374
2	*Alicyclobacillus acidiphilus*	AB076660	AAT**CC** GTTCCCGGGCCTT CTGAAACTCAAAGGAATTGACG	88%-	913-1374
3	*Alicyclobacillus acidoterrestris*	AB042057	AAT**CC** GTTCCCGGGCCTT CTGAAACTCAAAGGAATTGACG	88%+	910-1371
4	*Alicyclobacillus hesperidum*	AJ133633	AAT**CC** GTTCCCGGGCCTT CTGAAACTCAAAGGAATTGACG	88%+	884-1345
5	*Alicyclobacillus fastidiosus*	AB264021	AAT**C** CGTTCCCGGGCCTT CTGAAACTCAAAGGAATTGACG	88%+	910-1371
6	*Alicyclobacillus vulcanalis*	AY425985	AATACGTTCCCGGGCCTT CTGAAACTCAAAGGAATTGACG	88%+	894-1355
7	*Alicyclobacillus sendaiensis*	AB084128	AATACGTTCCCGGGCCTT CTGAAACTCAAAGGAATTGACG	88%+	885-1346
8	*Alicyclobacillus contaminans*	AB264026	AAT**C** CGTTCCCGGGCCTT CTGAAACTCAAAGGAATTGACG	88%+	925-1386
9	*Alicyclobacillus acidocaldarius* subsp. *acidocaldarius* (Type sp)	AJ496806	AATACGTTCCCGGGCCTT CTGAAACTCAAAGGAATTGACG	87%+	902-1363
10	*Alicyclobacillus aeris*	FM179383	AAT**CC** GTTCCCGGGCCTT CTGAAACTCAAAGGAATTGACG	88%+	911-1372
11	*Alicyclobacillus pomorum*	AB089840	AAT**C** CGTTCCCGGGCCTT CTGAAACTCAAAGGAATTGACG	88%+	911-1372
12	*Alicyclobacillus disulfidooxidans*	AB089843	AAT**C** CGTTCCCGGGCCTT CTGAAACTCAAAGGAATTGACG	85%+	911-1372
13	*Alicyclobacillus tolerans*	Z21979	AATACGTTCCCGGGCCTT CTGAAACTCAAAGGAATTGACG	87%-	906-1365
14	*Alicyclobacillus ferrooxydans*	EU137838	AAT**C** CGTTCCCGGGCCTT CTGAAACTCAAAGGAATTGACG	88%+	913-1374
15	*Alicyclobacillus cycloheptanicus*	AB042059	AAT**C** CGTTCCCGGGCCTT CTGAAACTCAAAGGAATTGACG	88%+	911-1372
16	*Alicyclobacillus macrosporangiidus*	AB264025	AATACGTTCCCGGGCCTT CTGAAACTCAAAGGAATTGACG	87%-	927-1388
17	*Alicyclobacillus kakegawensis*	AB264022	AAT**C** CGTTCCCGGGCCTT CTGAAACTCAAAGGAATTGACG	86%+	924-1385
18	*Alicyclobacillus shizuokensis*	AB264024	AAT**C** CGTTCCCGGGCCTT CTGAAACTCAAAGGAATTGACG	87%+	924-1385
19	*Alicyclobacillus herbarius*	AB042055	AAT**C** CGTTCCCGGGCCTT CTGAAACTCAAAGGAATTGACG	87%+	924-1385
20	*Alicyclobacillus pohliae*	AJ564766	AATACGTTCCCGGGCCTT CTGAAACTCAAAGGAATTGACG	88%+	904-1363
21	*Alicyclobacillus tolerans*	Z21979	AATACGTTCCCGGGCCTT CTGAAACTCAAAGGAATTGACG	87%-	906-1365
22	*Amphibacillus sediminis*	AB243866	AATACGTTCCCGGG**TC** TT CTGAAACTCAAA**A** GAATTGACG	96%-	928-1386
23	*Amphibacillus jilinensis,*	FJ169626	AATACGTTCCCGGG**TC** TT CTGAAACTCAAA**A** GAATTGACG	95%-	948-1406
24	*Amphibacillus tropicus*	AF418602	AATACGTTCCCGGG**TC** TT CTGAAACTCAAA**A** GAATTGACG	95%-	905-1362
25	*Amphibacillus fermentum*	AF418603	CTGAAACTCAAAGGAATTGACG AATACGTTCCCGGG**TC** TT	93%-	910-1368
26	*Amphibacillus xylanus,* type sp.	D82065	AATACGTTCCCGGG**TC** TT CTGAAACTCAAA**A** GAATTGACG	94%-	948-1406
27	*Aneurinibacillus aneurinilyticus* type sp.	X94194	AATACGTTCCCGGG**TC** TT CTGAAACTCAAAGGAATTGACG	91%-	903-1369
28	*Aneurinibacillus migulanus*	X94195	CTGAAACTCAAAGGAATTGACG AATACGTTCCCGGG**TC** TT	90%-	903-1359
29	*Aneurinibacillus danicus*	AB112725	CTGAAACTCAAAGGAATTGACG AATACGTTCCCGGG**TC** TT	91%-	903-1354
30	*Aneurinibacillus thermoaerophilus*	X94196	CTGAAACTCAAAGGAATTGACG AATACGTTCCCGGG**TC** TT	92%-	904- 1361
31	*Aneurinibacillus terranovensis*	AJ715385	CTGAAACTCAAAGGAATTGACG AATACGTTCCCGGG**TC** TT	91%-	897-1353
32	*Brevibacillus centrosporus*	D78458	**G** TGAAACTCAAAGGAATTGACG AATACGTTCCCGGGCCTT	91%+	917-1377
33	*Brevibacillus choshinensis*	AB112713	**G** TGAAACTCAAAGGAATTGACG AATACGTTCCCGGGCCTT	91%+	894-1354
34	*Brevibacillus reuszeri*	AB112715	**G** TGAAACTCAAAGGAATTGACG AATACGTTCCCGGGCCTT	91%+	894-1354
35	*Brevibacillus parabrevis*	AB112714	**G** TGAAACTCAAAGGAATTGACG AATACGTTCCCGGGCCTT	91%+	894-1354
36	*Brevibacillus brevis* type sp.	AB271756	**G** TGAAACTCAAAGGAATTGACG AATACGTTCCCGGGCCTT	91%+	896-1356
37	*Brevibacillus formosus*	AB112712	**G** TGAAACTCAAAGGAATTGACG AATACGTTCCCGGGCCTT	91%+	894-1354
38	*Brevibacillus agri*	AB112716	**G** TGAAACTCAAAGGAATTGACG AATACGTTCCCGGGCCTT	91%+	895-1355
39	*Brevibacillus limnophilus*	AB112717	**G** TGAAACTCAAAGGAATTGACG AATACGTTCCCGGGCCTT	91%+	909-1369
40	*Brevibacillus invocatus*	AF378232	CTGAAACTCAAAGGAATTGACG AATACGTTCCCGGGCCTT	91%+	896-1356
41	*Brevibacillus panacihumi*	EU383033	CTGAAACTCAAAGGAATTGACG AATACGTTCCCGGGCCTT	91%+	902-1362
42	*Brevibacillus borstelensis*	AB112721	**G** TGAAACTCAAAGGAATTGACG AATACGTTCCCGGGCCTT	92%+	894-1354
43	*Brevibacillus ginsengisoli*	AB245376	**G** TGAAACTCAAAGGAATTGACG AATACGTTCCCGGGCCTT	92%+	873-1333
44	*Brevibacillus laterosporus*	D16271	**G** TGAAACTCAAAGGAATTGACG AATACGTTCCCGGGCCTT	91%+	896-1356
45	*Brevibacillus fluminis*	EU375457	**G** TGAAACTCAAAGGAATTGACG AATACGTTCCCGGGCCTT	91%+	896-1356
46	*Brevibacillus levickii*	AJ715378	**G** TGAAACTCAAAGGAATTGACG AATACGTTCCCGGGCCTT	91%+	897-1357
47	*Brevibacillus thermoruber*	Z26921	**G** TGAAACTCAAAGGAATTGACG AATACGTTCCCGGGCCTT	92%+	915-1376
48	*Gracilibacillus lacisalsi*	DQ664540	CTGAAACTCAAA**A** GAATTGACG AATACGTTCCCGGGCCTT	94%+	933-1393
49	*Gracilibacillus thailandensis*	FJ182214	CTGAAACTCAAA**A** GAATTGACG AATACGTTCCCGGGCCTT	94%+	942-1402
50	*Gracilibacillus saliphilus*	EU784646	CTGAAACTCAAA**A** GAATTGACG AATACGTTCCCGGGCCTT	94%+	917-1377
51	*Gracilibacillus orientalis*	AM040716	CTGAAACTCAAA**A** GAATTGACG AATACGTTCCCGGGCCTT	93%+	931-1391
52	*Gracilibacillus dipsosauri*	AB101591	CTGAAACTCAAA**A** GAATTGACG AATACGTTCCCGGGCCTT	95%+	923-1383
53	*Gracilibacillus ureilyticus*	EU709020	CTGAAACTCAAA**A** GAATTGACG AATACGTTCCCGGGCCTT	95%+	923-1383
54	*Gracilibacillus boraciitolerans*	AB197126	CTGAAACTCAAA**A** GAATTGACG AATACGTTCCCGGGCCTT	94%+	935-1395
55	*Gracilibacillus halotolerans* type sp.	AF036922	CTGAAACTCAAA**A** GAATTGACG AATACGTTCCCGGGCCTT	94%+	934-1394
56	*Gracilibacillus halophilus*	EU135704	CTGAAACTCAAA**A** GAATTGACG AATACGTTCCCGGGCCTT	94%+	924-1384
57	*Paenibacillus polymyxa* type sp.	D16276	AATACGTTCCCGGG**T** CTT CTGAAACTCAAAGGAATTGACG	90%-	913-1375
58	*Paenibacillus antarcticus*	AJ605292	AATACGTTCCCGGG**T** CTT CTGAAACTCAAAGGAATTGACG	90%-	915-1374
59	*Paenibacillus macquariensis* subsp. *macquariensis*	X60625	AATACGTTCCCGGG**T** CTT CTGAAACTCAAAGGAATTGACG	90%-	935-1394
60	*Paenibacillus macquariensis* subsp. *defensor*	AB360546	AATACGTTCCCGGG**T** CTT CTGAAACTCAAAGGAATTGACG	90%+	936-1395
61	*Paenibacillus glacialis*	EU815294	AATACGTTCCCGGG**T** CTT CTGAAACTCAAAGGAATTGACG	91%-	934-1393
62	*Virgibacillus pantothenticus* type sp.	D16275	AATACGTTCCCGGG**TC** TT CTGAAACTCAAAGGAATTGACG	95%-	919-1375
63	*Virgibacillus proomii*	AJ012667	CTGAAACTC**AAAAGA** ATTGACG AATACGTTCCCGGG**TC** TT	95%-	916-1372
64	*Virgibacillus salexigens*	Y11603	CTGAAACTCAAA**AG** AATTNACG AATACGTTCCCGGGCCTT	95%+	921-1379
65	*Virgibacillus marismortui*	AJ009793	AATACGTTCCCGGGCCTT CTGAAACTCAAA**AG** AATTGACG	95%+	947-1407
66	*Virgibacillus salarius*	AB197851	AATACGTTCCCGGGCCTT CTGAAACTCAAA**A** GAATTGACG	95%+	949-1409
67	*Virgibacillus olivae*	DQ139839	AATACGTTCCCGGGCCTT CTGAAACTCAAA**AG** AATTGACG	95%+	948-1409
68	*Virgibacillus halodenitrificans*	AY543169,	CTGAAACTCAAA**AG** AATTGACG AATACGTTCCCGGGCCTT	95%+	926-1386
69	*Virgibacillus koreensis*	AY616012	CTGAAACTCAAA**AG** AATTGACG AATACGTTCCCGGGCCTT	97%+	926-1386
70	*Virgibacillus halophilus*	AB243851	CTGAAACTCAAA**AG** AATTGACG AATACGTTCCCGGGCCTT	94%+	926-1386
71	*Virgibacillus sediminis*	AY121430	CTGAAACTCAAA**AG** AATTGACG AATACGTTCCCGGGCCTT	96%-	946-1406
72	*Virgibacillus xinjiangensis*	DQ664543	CTGAAACTCAAA**AG** AATTGACG AATACGTTCCCGGGCCTT	96%-	894-1354
73	*Virgibacillus chiguensis*	EF101168	CTGAAACTCAAA**AG** AATTGACG AATACGTTCCCGGGCCTT	96%-	919-1375
74	*Virgibacillus dokdonensis*	AY822043	CTGAAACTCAAA**AG** AATTGACG AATACGTTCCCGGG**T** CTT	96%-	927-1383
75	*Virgibacillus carmonensis*	AJ316302	CTGAAACTCAAA**AG** AATTGACG AATACGTTCCCGGGCCTT	95%+	925-1385
76	*Virgibacillus necropolis*	AJ315056	CTGAAACTCAAA**AG** AATTGACG AATACGTTCCCGGGCCTT	95%+	925-1385
77	*Virgibacillus arcticus*	EF675742	CTGAAACTCAAA**AG** AATTGACG AATACGTTCCCGGGCCTT	95%+	809- 1269
78	*Virgibacillus byunsanensis*	FJ357159	CTGAAACTCAAA**AG** AATTGACG AATACGTTCCCGGGCCTT	95%+	923-1383
79	*Virgibacillus salinus*	FM205010	CTGAAACTCAAA**AG** AATTGACG AATACGTTCCCGGGCCTT	95%+	932-1392
80	*Virgibacillus subterraneus*	FJ746573	AATACGTTCCCGG**C** CCTT CTGAAACTCAAA**AG** AATTGACG	91%+	905-1362
81	*Virgibacillus kekensis*	AY121439	CTGAAACTCAAA**AG** AATTGACG AATACGTTCCCGGGCCTT	95%+	945-1405
82	*Geobacillus stearothermophilus* type sp.	AB021196,	CTGAAACTCAAAGGAATTGACG AATACGTTCCCGGGCCTT	93%+	912-1376
83	*Geobacillus kaustophilus*	X60618	CTGAAACTCAAAGGAATTGACG AATACGTTCCCGGGCCTT	92%+	933-1390
84	*Geobacillus lituanicus*	AY044055	CTGAAACTCAAAGGAATTGACG AATACGTTCCCGGGCCTT	92%+	933-1397
85	*Geobacillus thermoleovorans*	Z26923	CTGAAACTCAAAGGAATTGACG AATACGTTCCCGGGCCTT	93%+	918-1382
86	*Geobacillus thermocatenulatus*	AY608935	CTGAAACTCAAAGGAATTGACG AATACGTTCCCGGGCCTT	93%+	940-1404
87	*Geobacillus jurassicus*	AY312404	CTGAAACTCAAAGGAATTGACG AATACGTTCCCGGGCCTT	93%+	915-1379
88	*Geobacillus uzenensis*	AF276304	CTGAAACTCAAAGGAATTGACG AATACGTTCCCGGGCCTT	92%+	907-1370
89	*Geobacillus subterraneus*	AF276306	CTGAAACTCAAAGGAATTGACG AATACGTTCCCGGGCCTT	93%+	931-1395
90	*Geobacillus thermodenitrificans*	AY608961	CTGAAACTCAAAGGAATTGACG AATACGTTCCCGGGCCTT	93%+	939-1409
91	*Geobacillus debilis*	AJ564616	AATACGTTC**T** CGGGCCTT CTGAAACTCAAAGGAATTGACG	91%-	936-1398
92	*Geobacillus toebii*	AF326278	CTGAAACTCAAAGGAATTGACG AATACGTTCCCGGGCCTT	93%+	910-1374
93	*Geobacillus thermoglucosidasius*	AY608981	CTGAAACTCAAAGGAATTGACG AATACGTTCCCGGGCCTT	93%+	939-1405
94	*Geobacillus caldoxylosilyticus*	AF067651	CTGAAACTCAAAGGAATTGACG AATACGTTCCCGGGCCTT	94%+	925-1389
95	*Geobacillus tepidamans*	AY563003	**G** TGAAACTCAAAGGAATTGACG AATACGTTCCCGGGCCTT	95%+	872-1334
96	*Geobacillus vulcani*	AJ293805	CTGAAACTCAAAGGAATTGACG AATACGTTCCCGGGCCTT	93%+	920-1384
97	*Filobacillus milosensis*	AJ238042,	CTGAAACTCAAAGGAATTGACG AATACGTTCCCGGGCCTT	94%+	915-1375
98	*Jeotgalibacillus alimentarius.*	AF281158	CTGAAACTCAAAGGAATTGACG AATACGTTCCCGGGCCTT	96%+	911-1373
99	*Jeotgalibacillus salarius*	EU874389	CTGAAACTCAAAGGAATTGACG AATACGTTCCCGGGCCTT	96%+	910-1372
100	*Jeotgalibacillus campisalis*	AY190535	CTGAAACTCAAAGGAATTGACG AATACGTTCCCGGGCCTT	95%+	908-1370
101	*Jeotgalibacillus marinus*	AJ237708	CTGAAACTCAAAGGAATTGACG AATACGTTCCCGGGCCTT	95%+	918-1380
102	*Ureibacillus thermosphaericus*	AB101594	CTGAAACTCAAAGGAATTGACG AATACGTTCCCGGGCCTT	92%+	931-1392
103	*Ureibacillus composti*	DQ348071	CTGAAACTCAAAGGAATTGACG AATACGTTCCCGGGCCTT	92%+	929-1390
104	*Ureibacillus thermophilus*	DQ348072	CTGAAACTCAAAGGAATTGACG AATACGTTCCCGGGCCTT	91%+	931-1392
105	*Ureibacillus suwonensis*	AY850379	CTGAAACTCAAAGGAATTGACG AATACGTTCCCGGGCCTT	92%-	918-1379
106	*Ureibacillus terrenus*	AJ276403	CTGAAACTCAAAGGAATTGACG AATACGTTCCCGGGCCTT	92%-	900-1361
107	*Lysinibacillus boronitolerans*	AB199591	CTGAAACTCAAAGGAATTGACG AATACGTTCCCGGGCCTT	93%+	898-1360
108	*Lysinibacillus xylanilyticus*	FJ477040	CTGAAACTCAAAGGAATTGACG AATACGTTCCCGGGCCTT	93%+	826-1288
109	*Lysinibacillus fusiformis*	AJ310083	CTGAAACTCAAAGGAATTGACG AATACGTTCCCGGGCCTT	93%+	920-1382
110	*Lysinibacillus sphaericus*	AJ310084	CTGAAACTCAAAGGAATTGACG AATACGTTCCCGGGCCTT	93%+	920-1382
111	*Lysinibacillus parviboronicapiens*	AB300598	CTGAAACTCAAAGGAATTGACG AATACGTTCCCGGGCCTT	93%+	910-1372
112	*Terribacillus goriensis*	DQ519571	CTGAAACTCAAA**AG** AATTGACG AATACGTTCCCGGGCCTT	94%+	895-1355
113	*Terribacillus saccharophilus*	AB243845	CTGAAACTCAAA**AG** AATTGACG AATACGTTCCCGGGCCTT	94%+	922-1382
114	*Terribacillus halophilus*	AB243849	CTGAAACTCAAA**AG** AATTGACG AATACGTTCCCGGGCCTT	95%+	922-1382
115	*Terribacillus aidingensis*	FJ386524	CTGAAACTCAAA**AG** AATTGACG AATACGTTCCCGGGCCTT	95%+	922-1382
116	*Bacillus massiliensis*	AY677116	AATACGTTCCCGGGCCTT CTGAAACTCAAAGGAATTGACG	93%+	908-1370
117	*Bacillus cecembensis*	AM773821	AATACGTTCCCGGGCCTT CTGAAACTCAAAGGAATTGACG	93%+	931-1393
118	*Bacillus odysseyi*	AF526913	AATACGTTCCCGGGCCTT CTGAAACTCAAAGGAATTGACG	93%+	931-1393
119	*Bacillus decisifrondis*	DQ465405	AATACGTTCCCGGGCCTT CTGAAACTCAAAGGAATTGACG	85%+	843-1305
120	*Bacillus psychrodurans*	AJ277984	AATACGTTCCCGGGCCTT CTGAAACTCAAAGGAATTGACG	95%-	918-1380
121	*Bacillus psychrotolerans*	AJ277983	AATACGTTCCCGGGCCTT CTGAAACTCAAAGGAATTGACG	95%-	903-1365
122	*Bacillus insolitus*	AM980508	**GAGGGGTTCCCGGGCCTT** CTGAAACTCAAAGGAATTGACG	94%+	917-1378
123	*Bacillus beijingensis*	EF371374	**AATACGTTCCCGGGTCTT** CTGAAACTCAAAGGAATTGACG	96%-	929-1387
124	*Bacillus ginsengi*	EF371375	**AATACGTTCCCGGGTCTT** CTGAAACTCAAAGGAATTGACG	97%-	929-1387
125	*Bacillus aquimaris*	AF483625	AATACGTTCCCGGGCCTT CTGAAACTCAAAGGAATTGACG	99%+	910-1372
126	*Bacillus vietnamensis*	AB099708	AATACGTTCCCGGGCCTT CTGAAACTCAAAGGAATTGACG	98%+	903-1365
127	*Bacillus marisflavi*	AF483624	AATACGTTCCCGGGCCTT CTGAAACTCAAAGGAATTGACG	100%+	909-1371
128	*Bacillus seohaeanensis*	AY667495	AATACGTTCCCGGGCCTT CTGAAACTCAAAGGAATTGACG	98%+	872-1334
129	*Bacillus mycoides*	AB021192	AATACGTTCCCGGGCCTT CTGAAACTCAAAGGAATTGACG	97%+	907-1367
130	*Bacillus weihenstephanensis*	AB021199	AATACGTTCCCGGGCCTT CTGAAACTCAAAGGAATTGACG	97%+	925-1385
131	*Bacillus thuringiensis*	D16281	AATACGTTCCCGGGCCTT CTGAAACTCAAAGGAATTGACG	97%+	911-1371
132	*Bacillus pseudomycoides*	AF013121	CTGAAACTCAAAGGA**T** TTGACG AATACGTTCCCGGGCCTT	95%+	932-1392
133	*Bacillus funiculus*	AB049195	CTGAAACTCAAAGGAATTGACG AATACGTTCCCGGGCCTT	98%+	919-1379
134	*Bacillus panaciterrae*	AB245380	CTGAAACTCAAAGGAATTGACG AATACGTTCCCGGGCCTT	97%+	904-1364
135	*Bacillus flexus*	AB021185	CTGAAACTCAAAGGAATTGACG AATACGTTCCCGGGCCTT	98%+	923-1385
136	*Bacillus megaterium*	D16273	CTGAAACTCAAAGGAATTGACG AATACGTTCCCGGGCCTT	98%+	910-1372
137	*Bacillus koreensis*	AY667496	CTGAAACTCAAAGGAATTGACG AATACGTTCCCGGGCCTT	96%+	847-1309
138	*Bacillus aerius*	AJ831843	CTGAAACTCAAAGGAATTGACG AATACGTTCCCGGGCCTT	96%+	922-1382
139	*Bacillus aerophilus*	AJ831844	CTGAAACTCAAAGGAATTGACG AATACGTTCCCGGGCCTT	97%+	927-1387
140	*Bacillus stratosphericus*	AJ831841	CTGAAACTCAAAGGAATTGACG AATACGTTCCCGGGCCTT	97%+	927-1387
141	*Bacillus sonorensis*	AF302118	CTGAAACTCAAAGGAATTGACG AATACGTTCCCGGGCCTT	95%+	908-1368
142	*Bacillus amyloliquefaciens*	AB255669	CTGAAACTCAAAGGAATTGACG AATACGTTCCCGGGCCTT	95%+	909-1369
143	*Bacillus siamensis*	GQ281299	CTGAAACTCAAAGGAATTGACG AATACGTTCCCGGGCCTT	95%-	931-1352
144	*Bacillus methylotrophicus*	EU194897	CTGAAACTCAAAGGAATTGACG AATACGTTCCCGGGCCTT	95%+	898-1358
145	*Bacillus subtilis* subsp. *subtilis*	AJ276351	CTGAAACTCAAAGGAATTGACG AATACGTTCCCGGGCCTT	95%+	919-1379
146	*Bacillus subtilis* subsp. *spizizenii*	AF074970	CTGAAACTCAAAGGAATTGACG AATACGTTCCCGGGCCTT	95%+	907-1367
147	*Bacillus vallismortis*	AB021198	CTGAAACTCAAAGGAATTGACG AATACGTTCCCGGGCCTT	96%+	924-1384
148	*Bacillus mojavensis*	AB021191	CTGAAACTCAAAGGAATTGACG AATACGTTCCCGGGCCTT	96%+	920-1380
149	*Bacillus atrophaeus*	AB021181	CTGAAACTCAAAGGAATTGACG AATACGTTCCCGGGCCTT	96%+	909-1369
150	*Bacillus pumilus*	AY876289	CTGAAACTCAAAGGAATTGACG AATACGTTCCCGGGCCTT	97%+	879-1339
151	*Bacillus safensis*	AF234854	CTGAAACTCAAAGGAATTGACG AATACGTTCCCGGGCCTT	97%+	879-1339
152	*Bacillus altitudinis*	AJ831842	CTGAAACTCAAAGGAATTGACG AATACGTTCCCGGGCCTT	97%+	934-1394
153	*Bacillus ginsengihumi*	AB245378	**TT** GAAACTCAAAGGAATTGACG AATACGTTCCCGGGCCTT	98%-	914-1376
154	*Bacillus acidiproducens*	EF379274	**TT** GAAACTCAAAGGAATTGACG AATACGTTCCCGGGCCTT	97%-	879-1341
155	*Bacillus acidicola*	AF547209	CTGAAACTCAAAGGAATTGACG AATACGTTCCCGGGCCTT	99%+	934-1396
156	*Bacillus oleronius*	AY988598	CTGAAACTCAAAGGAATTGACG AATACGTTCCCGGGCCTT	97%+	934-1396
157	*Bacillus sporothermodurans*	U49078	CTGAAACTCAAAGGAATTGACG AATACGTTCCCGGGCCTT	97%+	904-1366
158	*Bacillus carboniphilus*	AB021182	CTGAAACTCAAAGGAATTGACG AATACGTTCCCGGGCCTT	95%+	910-1372
159	*Bacillus chungangensis*	FJ514932	CTGAAACTCAAAGGAATTGACG AATACGTTCCCGGGCCTT	94%+	890-1352
160	*Bacillus endophyticus*	AF295302	AATACGTTCCCGGG**TC** TT CTGAAACTCAAAGGAATTGACG	96%-	906-1362
161	*Bacillus isabeliae*	AM503357	CTGAAACTCAAAGGAATTGACG AATACGTTCCCGGGCCTT	98%+	912-1372
162	*Bacillus shackletonii*	AJ250318	CTGAAACTCAAAGGAATTGACG AATACGTTCCCGGGCCTT	98%+	909-1371
163	*Bacillus circulans*	AY043084	CTGAAACTCAAAGGAATTGACG AATACGTTCCCGGGCCTT	96%+	902-1364
164	*Bacillus nealsonii*	EU656111	CTGAAACTCAAAGGAATTGACG AATACGTTCCCGGGCCTT	97%+	928-1390
165	*Bacillus korlensis*	EU603328	CTGAAACTCAAAGGAATTGACG AATACGTTCCCGGGCCTT	98%+	889-1351
166	*Bacillus siralis*	AF071856	CTGAAACTCAAAGGAATTGACG AATACGTTCCCGGGCCTT	97%+	905-1367
167	*Bacillus benzoevorans*	X60611	AATACGTTCCCGGG**TC** TT CTGAAACTCAAAGGAATTGACG	95%-	931-1386
168	*Bacillus firmus*	D16268	CTGAAACTCAAAGGAATTGACG AATACGTTCCCGGGCCTT	97%+	907-1369
169	*Bacillus infantis*	AY904032	CTGAAACTCAAAGGAATTGACG AATACGTTCCCGGGCCTT	97%+	871-1333
170	*Bacillus oceanisediminis*	GQ292772	CTGAAACTCAAAGGAATTGACG AATACGTTCCCGGGCCTT	97%+	861-1323
171	*Bacillus kribbensis*	DQ280367	CTGAAACTCAAAGGAATTGACG AATACGTTCCCGGGCCTT	96%-	919-1381
172	*Bacillus horneckiae*	EU861362	**TT** GAAACTCAAAGGAATTGACG AATACGTTCCCGGGCCTT	98%+	796-1258
173	*Bacillus badius*	X77790	CTGAAACTCAAAGGAATTGACG AATACGTTCCCGGGCCTT	95%-	913-1370
174	*Bacillus smithii*	Z26935	CTGAAACTCAAAGGAATTGACG AATACGTTCCCGGGCCTT	95%+	924-1383
175	*Bacillus aeolius*	AJ504797	CTGAAACTCAAAGGAATTGACG AATACGTTCCCGGGCCTT	94%-	901-1361
176	*Bacillus coagulans*	AB271752	CTGAAACTCAAAGGAATTGACG AATACGTTCCCGGGCCTT	96%-	911-1373
177	*Bacillus alveayuensis*	AY605232	CTGAAACTCAAAGGAATTGACG AATACGTTCCCGGGCCTT	95%+	934-1396
178	*Bacillus thermoamylovorans*	L27478	CTGAAACTCAAAGGAATTGACG AATACGTTCCCGGGCCTT	92%+	930-1391
179	*Bacillus fordii*	AY443039	CTGAAACTCAAAGGAATTGACG AATACGTTCCCGGGCCTT	94%+	892-1354
180	*Bacillus fortis*	AY443038	CTGAAACTCAAAGGAATTGACG AATACGTTCCCGGGCCTT	94%+	927-1389
181	*Bacillus farraginis*	AY443036	CTGAAACTCAAAGGAATTGACG AATACGTTCCCGGGCCTT	94%+	838-1300
182	*Bacillus galactosidilyticus*	AJ535638	CTGAAACTCAAAGGAATTGACG AATACGTTCCCGGGCCTT	95%+	904-1367
183	*Bacillus ruris*	AJ535639	CTGAAACTCAAAGGAATTGACG AATACGTTCCCGGGCCTT	97%-	901-1363
184	*Bacillus lentus*	AB021189	CTGAAACTCAAAGGAATTGACG AATACGTTCCCGGGCCTT	97%+	928-1390
185	*Bacillus novalis*	AJ542512	CTGAAACTCAAAGGAATTGACG AATACGTTCCCGGGCCTT	97%+	908-1370
186	*Bacillus vireti*	AJ542509	CTGAAACTCAAAGGAATTGACG AATACGTTCCCGGGCCTT	96%+	908-1370
187	*Bacillus bataviensis*	AJ542508	CTGAAACTCAAAGGAATTGACG AATACGTTCCCGGGCCTT	98%+	908-1370
188	*Bacillus drentensis*	AJ542506	CTGAAACTCAAAGGAATTGACG AATACGTTCCCGGGCCTT	98%+	844-1306
189	*Bacillus soli*	AJ542513	CTGAAACTCAAAGGAATTGACG AATACGTTCCCGGGCCTT	97%+	908-1370
190	*Bacillus fumarioli*	AJ250056	CTGAAACTCAAAGGAATTGACG AATACGTTCCCGGGCCTT	95%+	909-1371
191	*Bacillus niacini*	AB021194	CTGAAACTCAAAGGAATTGACG AATACGTTCCCGGGCCTT	98%+	921-1383
192	*Bacillus pocheonensis*	AB245377	CTGAAACTCAAAGGAATTGACG AATACGTTCCCGGGCCTT	98%+	910-1372
193	*Bacillus boroniphilus*	AB198719	CTGAAACTCAAAGGAATTGACG AATACGTTCCCGGGCCTT	97%+	930-1392
194	*Bacillus selenatarsenatis*	AB262082	CTGAAACTCAAAGGAATTGACG AATACGTTCCCGGGCCTT	97%+	870-1332
195	*Bacillus jeotgali*	AF221061	CTGAAACTCAAAGGAATTGACG AATACGTTCCCGGGCCTT	97%+	908-1370
196	*Bacillus thioparans*	DQ371431	CTGAAACTCAAAGGAATTGACG AATACGTTCCCGGGCCTT	97%+	908-1370
197	*Bacillus foraminis*	AJ717382	CTGAAACTCAAAGGAATTGACG AATACGTTCCCGGGCCTT	97%+	922-1384
198	*Bacillus canaveralius*	DQ870688	CTGAAACTCAAAGGAATTGACG SEQUENEWAS SHORT	97%	887-1323
199	*Bacillus infernus*	U20385	CTGAAACTCAAAGGAATTGACG AATACGTTCCCGGGCCT**N**	95%+	921-1383
200	*Bacillus methanolicus*	AB112727	CTGAAACTCAAAGGAATTGACG AATACGTTCCCGGGCCTT	96%+	909-1372
201	*Bacillus butanolivorans*	EF206294	CTGAAACTCAAAGGAATTGACG AATACGTTCCCGGGCCTT	96%+	914-1380
202	*Bacillus simplex*	AJ439078	CTGAAACTCAAAGGAATTGACG AATACGTTCCCGGGCCTT	98%+	920-1379
203	*Bacillus muralis*	AJ316309	CTGAAACTCAAAGGAATTGACG AATACGTTCCCGGGCCTT	97%+	909-1371
204	*Bacillus psychrosaccharolyticus*	AB021195	CTGAAACTCAAAGGAATTGACG AATACGTTCCCGGGCCTT	96%+	900-1362
205	*Bacillus asahii*	AB109209	CTGAAACTCAAAGGAATTGACG AATACGTTCCCGGGCCTT	96%+	909-1373
206	*Bacillus indicus*	AJ583158	CTGAAACTCAAAGGAATTGACG AATACGTTCCCGGGCCTT	96%+	918-1381
207	*Bacillus cibi*	AY550276	CTGAAACTCAAAGGAATTGACG AATACGTTCCCGGGCCTT	97%+	896-1358
208	*Bacillus idriensis*	AY904033	CTGAAACTCAAAGGAATTGACG AATACGTTCCCGGGCCTT	97%+	889-1351
209	*Bacillus niabensis*	AY998119	CTGAAACTCAAAGGAATTGACG AATACGTTCCCGGGCCTT	95%+	904-1366
210	*Bacillus fastidiosus*	X60615	CTGAAACTCAAAGGAATTGACG AATACGTTCCCGGGCCTT	95%+	930-1386
211	*Bacillus litoralis*	AY608605	CTGAAACTCAAAGGAATTGACG AATACGTTCCCGGGCCTT	95%+	908-1370
212	*Bacillus herbersteinensis*	AJ781029	CTGAAACTCAAAGGAATTGACG AATACGTTCCCGGGCCTT	98%+	908-1370
213	*Bacillus galliciensis*	FM162181	CTGAAACTCAAAGGAATTGACG AATACGTTCCCGGGCCTT	96%+	908-1370
214	*Bacillus alkalitelluris*	AY829448	CTGAAACTCAAAGGAATTGACG AATACGTTCCCGGGCCTT	94%+	911-1373
215	*Bacillus humi*	AJ627210	CTGAAACTCAAAGGAATTGACG AATACGTTCCCGGGCCTT	96%	910-1372
216	*Bacillus halmapalus*	X76447	CTGAAACTCAAAGGAATTGACG AATACGTTCCCGGGCCTT	98%+	908-1370
217	*Bacillus horikoshii*	AB043865	CTGAAACTCAAAGGAATTGACG AATACGTTCCCGGGCCTT	97%+	929-1391
218	*Bacillus cohnii*	X76437	CTGAAACTCAAAGGAATTGACG AATACGTTCCCGGGCCTT	97%+	910-1372
219	*Bacillus acidiceler*	DQ374637	CTGAAACTCAAAGGAATTGACG AATACGTTCCCGGGCCTT	97%+	916-1376
220	*Bacillus luciferensis*	AJ419629	CTGAAACTCAAAGGAATTGACG AATACGTTCCCGGGCCTT	97%+	909-1369
221	*Bacillus azotoformans*	AB363732	CTGAAACTCAAAGGAATTGACG AATACGTTCCCGGGCCTT	97%+	909-1370
222	*Bacillus taeanensis*	AY603978	CTGAAACTCAAAGGAATTGACG AATACGTTCCCGGGCCTT	97%+	917-1378
223	*Bacillus macauensis*	AY373018	CTGAAACTCAAAGGAATTGACG AATACGTTCCCGGGCCTT	95%+	88-1350
224	*Bacillus rigui*	EU939689	CTGAAACTCAAAGGAATTGACG AATACGTTCCCGGGCCTT	96%-	903-1365
225	*Bacillus solisalsi*	EU046268	CTGAAACTCAAA**A** GGAATTGACG AATACGTTCCCGGGCCTT	95%-	887-1349
226	*Bacillus gelatini*	AJ551329	**T** TGAAACTCAAAGGAATTGACG AATACGTTCCCGGGCCTT	95%+	909-1371
227	*Bacillus arsenicus*	AJ606700	**T** TGAAACTCAAAGGAATTGACG AATACGTTCCCGGGCCTT	94%-	928-1390
228	*Bacillus barbaricus*	AJ422145	**T** TGAAACTCAAAGGAATTGACG AATACGTTCCCGGGCCTT	96%-	882-1342
229	*Bacillus algicola*	AY228462	**T** TGAAACTCAAAGGAATTGACG AATACGTTCCCGGGCCTT	97%+	931-1393
230	*Bacillus hwajinpoensis*	AF541966	**T** TGAAACTCAAAGGAATTGACG AATACGTTCCCGGGCCTT	96%+	909-1371
231	*Bacillus decolorationis*	AJ315075	**T** TGAAACTCAAAGGAATTGACG AATACGTTCCCGGGCCTT	94%+	909-1371
232	*Bacillus okuhidensis*	AB047684	**T** TGAAACTCAAAGGAATTGACG AATACGTTCCCGGGCCTT	96%+	874-1335
233	*Bacillus lehensis*	AY793550	AATACGTTCCCGGG**TC** TT CTGAAACTCAAAGGAATTGACG	95%-	939-1395
234	*Bacillus oshimensis*	AB188090	AATACGTTCCCGGG**TC** TT CTGAAACTCAAAGGAATTGACG	95%-	937-1393
235	*Bacillus patagoniensis*	AY258614	AATACGTTCCCGGG**TC** TT **T** TGAAACTCAAAGGAATTGACG	95%-	913-1369
236	*Bacillus clausii*	X76440	AATACGTTCCCGGG**TC** TT CTGAAACTCAAAGGAATTGACG	95%-	913-1369
237	*Bacillus gibsonii*	X76446	CTGAAACTCAAAGGAATTGACG AATACGTTCCCGGGCCTT	95%+	912-1372
238	*Bacillus murimartini*	AJ316316	CTGAAACTCAAAGGAATTGACG AATACGTTCCCGGGCCTT	95%+	913-1373
239	*Bacillus plakortidis*	AJ880003	CTGAAACTCAAAGGAATTGACG AATACGTTCCCGGGCCTT	95%+	906-1366
240	*Bacillus pseudalcaliphilus*	X76449	CTGAAACTCAAAGGAATTGACG AATACGTTCCCGGGCCTT	96%+	909-1371
241	*Bacillus trypoxylicola*	AB434284	CTGAAACTCAAAGGAATTGACG AATACGTTCCCGGGCCTT	97%+	911-1373
242	*Bacillus alcalophilus*	X76436	CTGAAACTCAAAGGAATTGACG AATACGTTCCCGGGCCTT	96%+	909-1371
243	*Bacillus bogoriensis*	AY376312	CTGAAACTCAAAGGAATTGA**GC** AATACGTTCCCGGGCCTT	97%+	911-1374
244	*Bacillus akibai*	AB043858	**T** TGAAACTCAAAGGAATTGACG AATACGTTCCCGGGCCTT	96%+	950-1411
245	*Bacillus krulwichiae*	AB086897	**T** TGAAACTCAAAGGAATTGACG AATACGTTCCCGGGCCTT	94%+	912-1374
246	*Bacillus okhensis*	DQ026060	**T** TGAAACTCAAAGGAATTGACG AATACGTTCCCGGGCCTT	96%+	916-1378
247	*Bacillus wakoensis*	AB043851	**T** TGAAACTCAAAGGAATTGACG AATACGTTCCCGGGCCTT	95%+	930-1392
248	*Bacillus hemicellulosilyticus*	AB043846	**T** TGAAACTCAAAGGAATTGACG AATACGTTCCCGGGCCTT	96%+	940-1402
249	*Bacillus macyae*	AY032601cpf	**T** TGAAACTCAAAGGAATTGACG AATACGTTCCCGGGCCTT	96%+	916-1378
250	*Bacillus alkalinitrilicus*	EF422411	CTGAAACTCAAAGGAATTGACG AATACGTTCCCGGGCCTT	95%+	919-1381
251	*Bacillus pseudofirmus*	X76439	**T** TGAAACTCAAAGGAATTGACG AATACGTTCCCGGGCCTT	97%+	910-1372
252	*Bacillus qingdaonensis*	DQ115802	**T** TGAAACTCAAAGGAATTGACG AATACGTTCCCGGGCCTT	93%+	913-1375
253	*Bacillus halochares*	AM982516	**T** TGAAACTCAAAGGAATTGACG AATACGTTCCCGGGCCTT	93%-	881-1343
254	*Bacillus aidingensis*	DQ504377	**T** TGAAACTCAAAGGAATTGACG AATACGTTCCCGGGCCTT	93%-	946-1407
255	*Bacillus salarius*	AY667494	**T** TGAAACTCAAAGGAATTGACG AATACGTTCCCGGGCCTT	93%+	858-1320
256	*Bacillus persepolensis*	FM244839	**T** TGAAACTCAAAGGAATTGACG AATACGTTCCCGGGCCTT	93%+	940-1402
257	*Bacillus agaradhaerens*	X76445	CTGAAACTCAAAGGAATTGACG AATACGTTCCCGGG**T** CTT	96%+	925-1385
258	*Bacillus neizhouensis*	EU925618	**T** TGAAACTCAAAGGAATTGACG AATACGTTCCCGGGCCTT	96%+	905-1367
259	*Bacillus beveridgei*	FJ825145	CTGAAACTCAAAGGAATTGACG AATACGTTCCCGGGCCTT	94%+	944-1409
260	*Bacillus chagannorensis*	*AM492159*	CTGAAACTCAAAGGAATTGACG AATACGTTCCCGGGCCTT	94%+	945-1407
261	*Bacillus saliphilus*	AJ493660	CTGAAACTCAAAGGAATTGACG AATACGTTCCCGGGCCTT	93%+	919-1381
262	*Bacillus aurantiacus*	AJ605773	CTGAAACTCAAAGGAATTGACG AATACGTTCCCGGGCCTT	95%+	929-1381
263	*Bacillus vedderi*	Z48306	CTGAAACTCAAAGGAATTGACG AATACGTTCCCGGGCCTT	95%-	905-1367
264	*Bacillus cellulosilyticus*	AB043852	CTGAAACTCAAAGGAATTGACG AATACGTTCCCGGGCCTT	95%-	924-1386
265	*Bacillus clarkii*	X76444	CTGAAACTCAAAGGAATTGACG AATACGTTCCCGGGCCTT	95%+	926-1328
266	*Bacillus polygoni*	AB292819	CTGAAACTCAAAGGAATTGACG AATACGTTCCCGGGCCTT	94%-	945-1408
267	*Bacillus horti*	D87035	CTGAAACTCAAAGGAATTGACG AATACGTTCCCGGGCCTT	93%+	923-1378
268	*Bacillus mannanilyticus*	AB043864	AATACGTTCCCGGG**TC** TT CTGAAACTCAAAGGAATTGACG	96%-	955-1413
	Actinobacteria (High GC content gram positive bacteria)				
269	*Corynebacterium diphtheriae*	X84248	CT**A** AAACTCAAAGGAATTGACG AATACGTNCCCGGGCCTT	83%-	880-1341
270	*Mycobacterium tuberculosis*	X58890	CTAAAACTCAAAGGAATTGACG AATACGTTCCCGGGCCTT	85%-	1541-2002
271	*Nocardia asteroides*	AF430019	CT**A** AAACTCAAAGGAATTGACG AATACGTTCCCGGGCCTT	84%-	875-1376
272	*Streptomyces lavendulae* subsp. *lavendulae*	D85116	CT**A** AAACTCANAGGAATTGACG AATACGTTCCCGGGCCTT	81%-	893-1361
	Low GC content Firmicutes (gram +ve)				
273	*Staphylococcus chromogenes*	D83360	AATACGTTCCCGGG**TC** TT CTGAAACTCAAAGGAATTGACG	92%+	913-1371
274	*Streptococcus pyogenes*	AB002521	**T** TGAAACTCAAAGGAATTGACG AATACGTTCCCGGGCCTT	89%+	890-1350
275	*Enterococcus faecalis*	AB012212	**T** TGAAACTCAAAGGAATTGACG AATACGTTCCCGGGCCTT	91%+	939-1395
276	*Clostridium populeti*	X71853	**A** TGAAACTCAAAGGAATTGACG AATACGTTCCCGGG**TC** TT	86%-	903-1359
277	*Listeria monocytogenes.*	*X56153*	AATACGTTCCCGGGCCT**N T** TGAAACTCAAAGGAATTGACG	94%+	936-1392
	Alpha proteobacteria				
278	*Rhizobium leguminosarum*	U29386	**TTA** AAACTCAAAGGAATTGACG AATACGTTCCCGGGCCTT	86%-	913-1371
279	*Azospirillum lipoferum*	Z29619	**TTA** AAACTCAAAGGAATTGACG AATACGTTCCCGGGCCTT	84%-	845-1305
280	*Acetobacterium woodii*	X96954	**T** TGAAACTCAAAGGAATTGACG AAT**G** CGTTCCCGGGTCTT	90%-	840-1305
	Beta proteobacteria				
281	*Burkholderia cepacia*	U96927	AATACGTTCCCGGG**TC** TT T**TA** AAACTCAAAGGAATTGACG	82%-	870-1322
282	*Bordetella pertussis*	U04950	**TTA** AAACTCAAAGGAATTGACG AATACGTTCCCGGG**TC** TT	81%-	922-1375
	Gamma proteobacteria				
283	*Pseudomonas aeruginosa*	X06684	AATACG**TC** CCCGGGCCTT **TTA** AAACTCAAATGAATTGACG	86%-	923-1384
284	*Escherichia coli*	X80725	**TTA** AAACTCAAATGAATTGACG AATACGTTCCCGGG**TC** TT	83%-	921-1379
285	*Klebsiella pneumoniae* subsp. *pneumoniae*	X87276	**TT** AAAACTCAAATGAATTGACG AATACGTTCCCGGG**TC** TT	82%-	920-1381
286	*Shigella dysenteriae*	X96966	**TTA** AAACTCAAATGAATTGACG AATACGTTCCCGGG**TC** TT	83%-	908-1362

### Multiple alignments of 463 bp sequences of different strains of Bacilli

**Figure 2:**
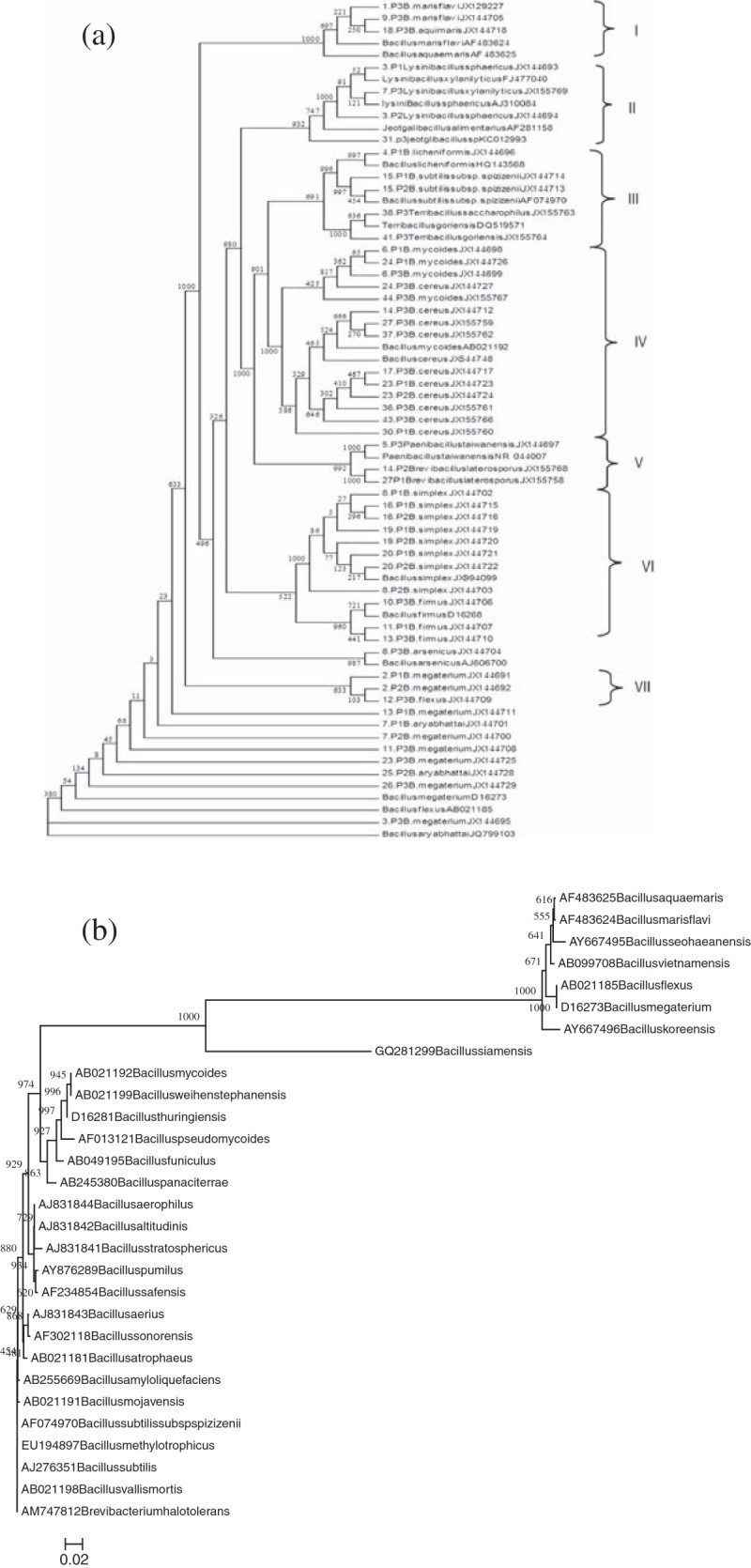
**Dendrograms showing the phylogenetic relationship (a) 52 AEFB strains with reference sequences (b) 29 closely related species of genera** ***Bacillus*** **based on 463 bp long 16S rRNA sequences.**

Dendrogram prepared on the basis of alignment of 463 bp sequence has been given in Figure 2(a&b). Dendrogram prepared for 52 different strains of *Bacillus* and related genera (taken in our study) and some reference sequences downloaded from NCBI has been shown in Figure 2(a). Dendrogram has been divided in to 7 different groups (I-VII). Group I contains strains belonging to species *Bacillus aquimaris* and *marisflavi*. Strains belonging to genera *Lysinibacillus* (*sphaericus* and *xylanilyticus*) and *Jeotgalibacillus* are present in Group II. Group III contains strains belonging to Genera *Terribacillus* (*sacharrophilus* and *goriensis*), *Bacillus subtilis* sub sp. *spizizinii* and *Bacillus licheniformis*. Group IV contains strains belonging to species, *Bacillus mycoides* and *Bacillus cereus*. Group V contains strains belonging to genera *Paenibacillus* and *Brevibacillus* and strains belonging to species *Bacillus simplex* and *Bacillus firmus* have shared the group VI. *Bacillus arsenicus* has not shown any grouping with any other species or genera and *Bacillus megaterium* and *Bacillus flexus* have shared a single group VII while some strains of *Bacillus megaterium*, *Bacillus flexus* and *Bacillus aryabhattai* have not shown any grouping with any other strain. Second dendrogram (Figure 2b) containing 29 different closely related species has been divided in to two major clusters and only one species *Bacillus siamensis* GQ281299 has not shown any grouping with any other member. 7 bacterial species i.e. *Bacillus aquaemaris* AF483625, *Bacillus marisflavi* AF483624, *Bacillus seohaeanensis* AY667495, *Bacillus vietnamensis* AB099708, *Bacillus flexus* AB021185, *Bacillus megaterium* D16273, *Bacillus koreensis* AY667496 lie in one cluster. Other, 21 bacterial strains have shared the other major cluster.

## Discussion

When we use molecular approaches to study microbial communities then the use of universal primers is not fully successful in finding the clear picture of community. Various researchers have faced such type of problems as Van Elsas et al. (2002) faced the problem when they studied two 16S rDNA clone libraries (one from grassland and one from arable land) prepared with bacterial primers and most of the isolated strains were found to be belonging to phylum Proteobacteria and the number of clones related to Bacilli were very few. When the same microbial communities were studied by Garbeva et al. (2003), by using *Bacillus* specific primers then a lot of *Bacillus* clones were isolated. The problem faced by universal primers can be overcome by the use of group specific primers and various researchers have used group specific primers in their studies to overcome this problem. Heuer and Smalla (1997) used Actinomycete specific primers to monitor Actinomycete communities in the potato rhizosphere. Similarly Boon et al. (2001) used several group specific nested PCR systems to identify a lot of groups under same DGGE conditions. So the need of group specific primers is there to find out the diversity and identity of the members of a specific group.

In the present research we have focused on identification and classification of AEFB by using a specific fragment of 16S rRNA gene. So in the following session we have discussed the research related to identification and classification of bacilli by using 16S rRNA gene. As Many researchers have developed a lot of different *Bacillus* specific primers i.e. Goto et al. (2000), synthesized a *Bacillus* specific prime pair which was used to amplify a 275 bp sequence near the 5’ end of 16S rDNA gene and this sequence was very specific for identification and classification of *Bacillus* strains. Garbeva et al. (2003) developed a *Bacillus* specific primer pair (Bac F and Bac R). Specificity of both primers was checked independently and some species of *Bacillus* and other related genera have shown 100% similarity with primer Bac F and likewise the reverse primer has shown similarity with 31 different species of *Bacillus* and related genera. Vardhan et al. (2011) developed a primer pair specific for amplification of a hyper variable region in 16S rDNA gene of *Bacillus* and related genera.

In the present study we found that a restriction digestion product of 16S rRNA gene (460 bp) by HaeIII enzyme was specific for *Bacillus* and related genera. Position of this fragment was near the 3’ end of 16S rDNA gene and primer pair specific to this 463 bp fragment has been designed. Primer pair when checked for specificity has shown amplification of a 463 bp long fragment in strains belonging to genera *Bacillus, Lysinibacillus, Terribacillus, Brevibacillus* and *Jeotgalibacillus*. No any amplification was seen in two AEFB strains i.e. *Bacillus arsenicus* and *Paenibacillus taiwanensis* and 9 different strains of bacterial lineages other than AEFB (Figure 1c&d). Reason for no amplification of this fragment in *Bacillus arsenicus* and *Paenibacillus taiwanensis* is may be due to the reason that during the course of evolution these have faced some variations because of which the restriction enzyme sites for Hae III enzymes were deleted at that position and primer pair designed in the present study includes the restriction site which causes the non specificity for primer.

Results of our study indicate that primer pair designed here is specific for *Bacillus* and related genera and not for other bacterial lineages. Primer pair when checked for homology (*in silico*) has shown 100% homology with 16S rDNA sequences of 120 species related to genera *Bacillus. Bacillus* species which do not have shown 100% similarity of these primers have acquired anomalous positions in the classification based on 16S rRNA gene (Yarza et al. 2010). While some species i.e. *B. pseudomycoides* AF013121, *B. ginsengihumi* AB245378*, B. acidiproducens* EF379274*, B. endophyticus,* AF295302*, B. benzoevorans,* X60611*, B. horneckiae* EU86136 have shown anomalous positions with other bacterial lineages according to classification systems based on 16S rRNA gene (Yarza et al. 2010) in spite of having homology with primer pair in our study. Bacilli strains other than the genus *Bacillus* have also shown the primer pair similarity and these genera are *Virgibacillus* (7)*, Geobacillus* (5), *Filobacillus* (1), *Jeotgalibacillus* (4) and *Ureibacillus* (5). Almost all the species checked for primer pair homology has shown 100% similarity except *Virgibacillus pantothenticus* D16275, *Virgibacillus proomii* and AJ012667. All of these genera belong to the family Bacillaceae except *Jeotgalibacillus* which belong to the family Planococcaceae. *Bacillus* related genera which don’t have shown primer pair similarity are *Alicyclobacillus* (7), *Amphibacillus* (5), *Aneurinibacillus* (5), *Brevibacillus* (16), *Gracilibacillus* (9) *and Paenibacillus* (5). Only a few members of these genera have shown homology with primer pair and these are *Alicyclobacillus acidocaldarius* AJ496806, *Alicyclobacillus tolerans* Z21979, *Brevibacillus invocatus* AF378232, *Brevibacillus panacihum.* Genera which do not have shown primer specificity belong to different species other than Bacillaceae except *Amphibacillus*, *Gracilibacillus* and *Terribacillus*. In our study genera belonging to family Bacillaceae have shown primer specificity and genera belonging to family other than Bacillaceae have not shown primer specificity except some genera which have shown primer specificity in reverse order.

Phylogenetic relationship based on 463 bp sequence of 52 bacilli strains (taken in our study) along with reference sequences (downloaded from NCBI) (Figure 2a) has shown that different bacterial strains belonging to same species and genera have shared a single group except some strains belonging to *Bacillus megaterium*, *B. aryabhattai* and *B. flexus*. As strains belonging to species *Bacillus megaterium* have not grouped in one cluster. Out of total 8 strains of *B. megaterium*, only two strains belonging to species *B. megaterium* have made grouping with *B. flexus*. Another 6 strains of *B. megaterium* and two strains of *B. aryabhattai* have not shown any grouping with any other strain, however all these eight strains lie below *B. megaterium and B. flexus* group. This shows that different strains of *B. megaterium* and *B.* aryabhattai (close relative of *B. megaterium*) have remarkable strain to strain genetic variations. Grouping of strains belonging to *Bacillus* related genera in between the strains related to *Bacillus* indicates that during the course of evolution these genera have been evolved from the older one genera i.e. *Bacillus* which is similar to the classifications according to others (Xu and Cote 2003; Yarza et al. 2010; Vardhan et al. 2011). Further the phylogenetic relationship of some closely related strains of genera *Bacillus*, sharing a single cluster in the all species living tree (Yarza et al. 2010) have shown the same phylogenetic relationship in our study (Figure 2b). The only exception is *Bacillus siamensis* GQ281299 which has not shown any grouping with any other *Bacillus* species. However, in all species living tree this strain has shown relationship with other *Bacillus* species which lie in the lower cluster in our study (Figure 2b).

From the present study we can conclude that the restriction digestion of 16S rRNA gene by HaeIII enzyme and amplification of 463 bp fragment with specific primers designed in our study are easy methods for identification of *Bacillus* and related genera. Further the sequence information and multiple alignment of 463 bp fragment of *Bacillus* and related genera have been proved to be a good identification and classification tool for *Bacillus* and related genera.

## Authors’ original submitted files for images

Below are the links to the authors’ original submitted files for images. Authors’ original file for figure 1 Authors’ original file for figure 2 Authors’ original file for figure 3

## References

[CR1] Ash C, Farrow AE, Wallbanks S, Collins MD (1991). Phylogenetic heterogeneity of the genus *Bacillus* revealed by comparative analysis of small-subunit-ribosomal RNA sequences. Lett Appl Microbiol.

[CR2] Ash C, Priest FG, Collins D (1993). Molecular identification of rRNA group 3 bacilli (Ash, Farrow, Wallbanks and Collins) using a PCR probe test. Proposal for the creation of a new genus *Paenibacillus*. Antonie van Leeuwenhoek.

[CR3] Boon N, Windt W, Verstraete W, Top EM (2001). Evaluation of nested PCR-DGGE (denaturing gradient gel electropho-resis) with group-specific 16S rRNA primers for the analysis of bacterial communities from different waste water treatment plants. FEMS Microbiol Ecol.

[CR4] Daffonchio D, Borin S, Consolandi A, Mora D, Manachini PL (1998). 16S–23S rRNA internal transcribed spacers as molecular markers for the species of the 16S rRNA group I of the genus *Bacillus*. FEMS Microbiol Lett.

[CR5] Daffonchio D, Borin S, Frova G, Manachini PL, Sorlini C (1998). PCR fingerprinting of whole genomes: the spacers between the 16S and 23S rRNA genes and of intergenic tRNA gene regions reveal a different intraspecific genomic variability of *Bacillus cereus* and *Bacillus licheniformis*. Int J Syst Bacteriol.

[CR6] De Clerck E, Van Mol K, Jannes G, Rossau R, de Vos P (2004). Design of a 50 exonuclease-based real-time PCR assay for simultaneous detection of *Bacillus licheniformis*, members of the '*B. cereus* group’ and B. *fumarioli* in gelatin. Lett Appl Microbiol.

[CR7] Fortina MG, Pukall R, Schumann P, Mora D, Parini C (2001). *Ureibacillus* gen. nov., a new genus to accommodate *Bacillus thermosphericus* (Anderson et al. 1995) emendation of *Ureibacillus thermosphericus* and description of *Ureibacillus terrreneus* sp. nov. Int J Syst Evol Microbiol.

[CR8] Garbeva P, van Veen JA, van Elsas JD (2003). Predominant *Bacillus* spp. in Agricultural Soil under Different Management Regimes Detected via PCR-DGGE. Microb Ecol.

[CR9] Goto K, Omura T, Hara Y, Sadaie Y (2000). Application of the partial 16S rDNA sequence as an index for rapid identification of the species in the genus *Bacillus*. J Gen Appl Microbiol.

[CR10] Gurtler V, Stanisich VA (1996). New approaches for typing and identification of bacteria using the 16S–23S rDNA spacer region. Microbiology.

[CR11] Heuer H, Smalla K, Van Elsas JD, Trevors JT, Wellington EMH (1997). Application of denaturing gradient gel electrophoresis (DGGE) and temperature gradient gel electrophoresis (TGGE) for studying soil microbial com-munities. Modern Soil Microbiology.

[CR12] Heyndrickx M, Lebbe L, Kersters K, Devos P, Forsyth G (1998). *Virgibacillus*: a new genus to accommodate *Bacillus pantothenticus* (Proom and Knight 1950). Emended description of *Virgibacillus pantothenticus*. Int J Syst Bacteriol.

[CR13] Kadyan S, Panghal M, Kumar S, Singh K, Yadav JP (2013). Assessment of functional and genetic diversity of aerobic endospore forming Bacilli from rhizospheric soil of *Phyllanthus amarus* L. World J Microbiol Biotechnol.

[CR14] Kumar P, Khare S, Dubey RC (2012). Diversity of Bacilli from Disease Suppressive Soil and their Role in Plant Growth Promotion and Yield Enhancement. New York Sci J.

[CR15] Larkin MA, Blackshields G, Brown NP, Chenna R, McGettigan PA (2007). Clustal W and Clustal X version 2.0. Bioinformatics.

[CR16] Mandic-Mulec I, Prosser JI, Logan NA, De Vos P (2011). Diversity of Endospore-forming Bacteria in Soil: Characterization and Driving Mechanisms. Endospore-forming Soil Bacteria, Soil Biology 27.

[CR17] Nazina TN, Tourova TP, Poltaraus AB, Novikova EV, Grigoryan AA (2001). Taxonomic study of aerobic thermophilic bacilli: descriptions of *Geobacillus subterraneus* gen. nov. sp. nov. and *Geobacillus uzenensis* sp. nov. from petroleum reservoirs and transfer of *Bacillus stearothermophilus*, *Bacillus thermocatenulatus*, *Bacillus thermoleovorans*, *Bacillus kaustophilus*, *Bacillus thermoglucosidasius*, *Bacillus thermodenitrificans* to *Geobacillus* as *Geobacillus stearothermophilus*, *Geobacillus thermocatenulatus*, *Geobacillus thermoleovorans*, *Geobacillus kaustophilu*, *Geobacillus thermoglucosidasius*, *Geobacillus thermodenitrificans*. Int J Syst Evol Microbiol.

[CR18] Niimura Y, Koh E, Yanagida F, Suzuki KI, Komagata K (1990). *Amphibacillus xylanus* gen. nov., a facultatively anaerobic spore foming xylin-digesting bacterium which lacks cytochrome, quinone, and catalase. Int J Syst Bacteriol.

[CR19] Priest FG, Goodfellow M, Todd C (1988). A numerical classification of the genus *Bacillus*. J Gen Microbiol.

[CR20] Schlesner H, Lawson PA, Collins MD, Weiss N, Wehmeyer U (2001). *Filobacillus milensis* gen. nov., a new halophilic spore-forming bacterium with Ornd- Glu-type peptidoglycan. Int J Syst Microbiol.

[CR21] Shida O, Takagi H, Kadowaki K, Yano H, Komagata K (1996). Proposal for two new genera, *Brevibacillus* gen. Nov. and *Aneurinibacillus* gen. nov. Int J Syst Bacteriol.

[CR22] Stackebrandt E, Swiderski J, Berkeley R, Heyndrickx M, Logan N, De Vos P (2002). From phylogeny to systematics: The dissection of the genus *Bacillus*. Applications and systematics of Bacillus and relatives.

[CR23] Tamura K, Peterson D, Peterson N, Stecher G, Nei M, Kumar S (2011). MEGA5: Molecular Evolutionary Genetics Analysis Using Maximum Likelihood, Evolutionary Distance and Maximum Parsimony Method. Mol Biol Evol.

[CR24] Van Elsas JD, Garbeva P, Salles J (2002). Effects of agricultural measures on the microbial diversity of soil as related to suppression of soil-borne plant pathogens. Biodegradation.

[CR25] Vardhan S, Kaushik R, Saxena AK, Arora DK (2011). Restriction analysis and partial sequencing of the 16S rRNA gene as index for rapid identification of *Bacillus* species. Antonie Van Leeuwenhoek.

[CR26] Waino M, Tindall BJ, Schumann P, Ingvorsen K (1999). *Gracillbacillus* gen. nov., with description of *Graclibacillus holotolerence* gen. nov., and *Bacillus salexigens* to the genus *Salibacillus* gen. nov., as *Salibacillus salexigens* comb. Nov. Int J syst bacteriol.

[CR27] Wisotzkey JD, Jurtshuk P, Fox GE, Deinhard G, Poralla K (1992). Comparative sequence analyses on the 16S r RNA (rDNA) of *Bacillus acidocaldarius*, *Bacillus acidoterrestris*, and *Bacillus cycloheptanicus* and proposal for certain of a new genus, *Alicyclobacillus* gen. nov. Int J Syst Bacteriol.

[CR28] Xu D, Cote JC (2003). Phylogenetic relationships between *Bacillus* species and related genera inferred from comparison of 3’ end 16S rDNA and 5’ end 16S–23S ITS nucleotide sequences. Int J Syst Evol Microbiol.

[CR29] Yarza PW, Ludwig J, Euzeby R, Amann KH, Schleifer FO (2010). Update of the All-Species Living Tree Project based on 16S and 23S rRNA sequence analyses. Syst Appl Microbiol.

[CR30] Yoon JH, Weiss N, Lee KC, Lee IS, Kang KH (2001). *Jeotgalibacillus alimentarius* gen. nov., sp. Nov., a novel bacterium isolated from jeotgal with L-lysine in the cell wall, reclassification of *Bacillus marinus* (Ruger 1983) as *Marinibacillus marinus* gen. nov., comb. Nov. Int J Syst Evol Microbiol.

