# Supplementary figures and images for: Development of a PCR based marker system for easy identification and classification of aerobic endospore forming bacilli
Source: Springerplus. 2013 Nov 9;2(1):596. doi: 10.1186/2193-1801-2-596 (PMC3840746; doi:10.1186/2193-1801-2-596)

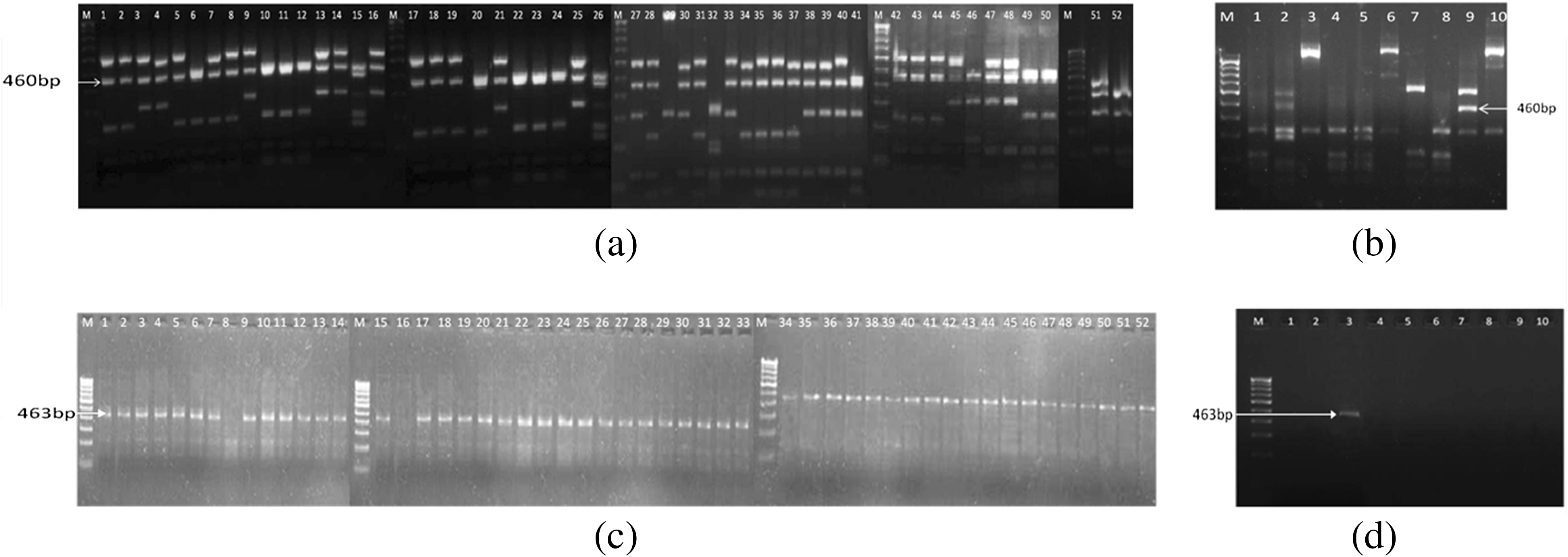

Supplement: Supplementary file 1 — Authors’ original file for figure 1 [file 40064_2013_657_MOESM1_ESM.tif]

(a)

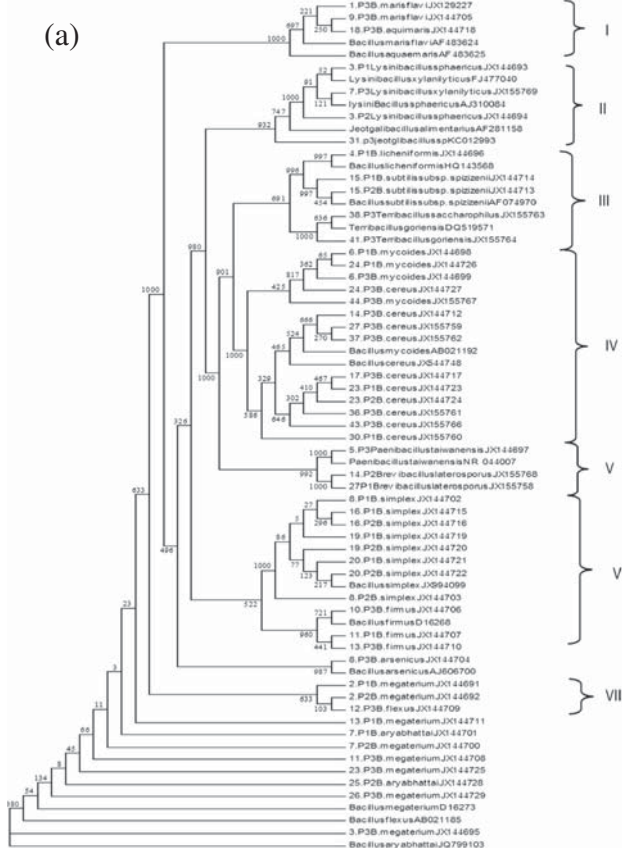

(b)

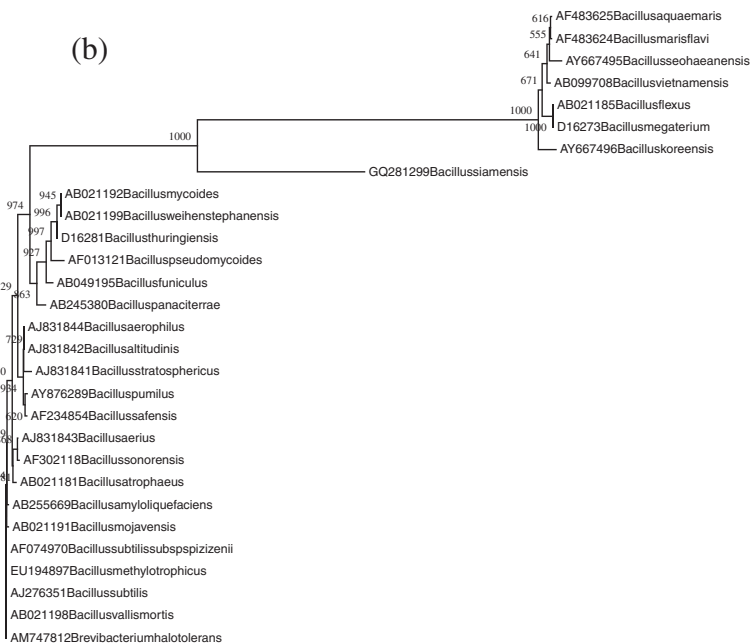

0.02

Supplement: Supplementary file 2 — Authors’ original file for figure 2 [file 40064_2013_657_MOESM2_ESM.pdf]

Fig 3(a)


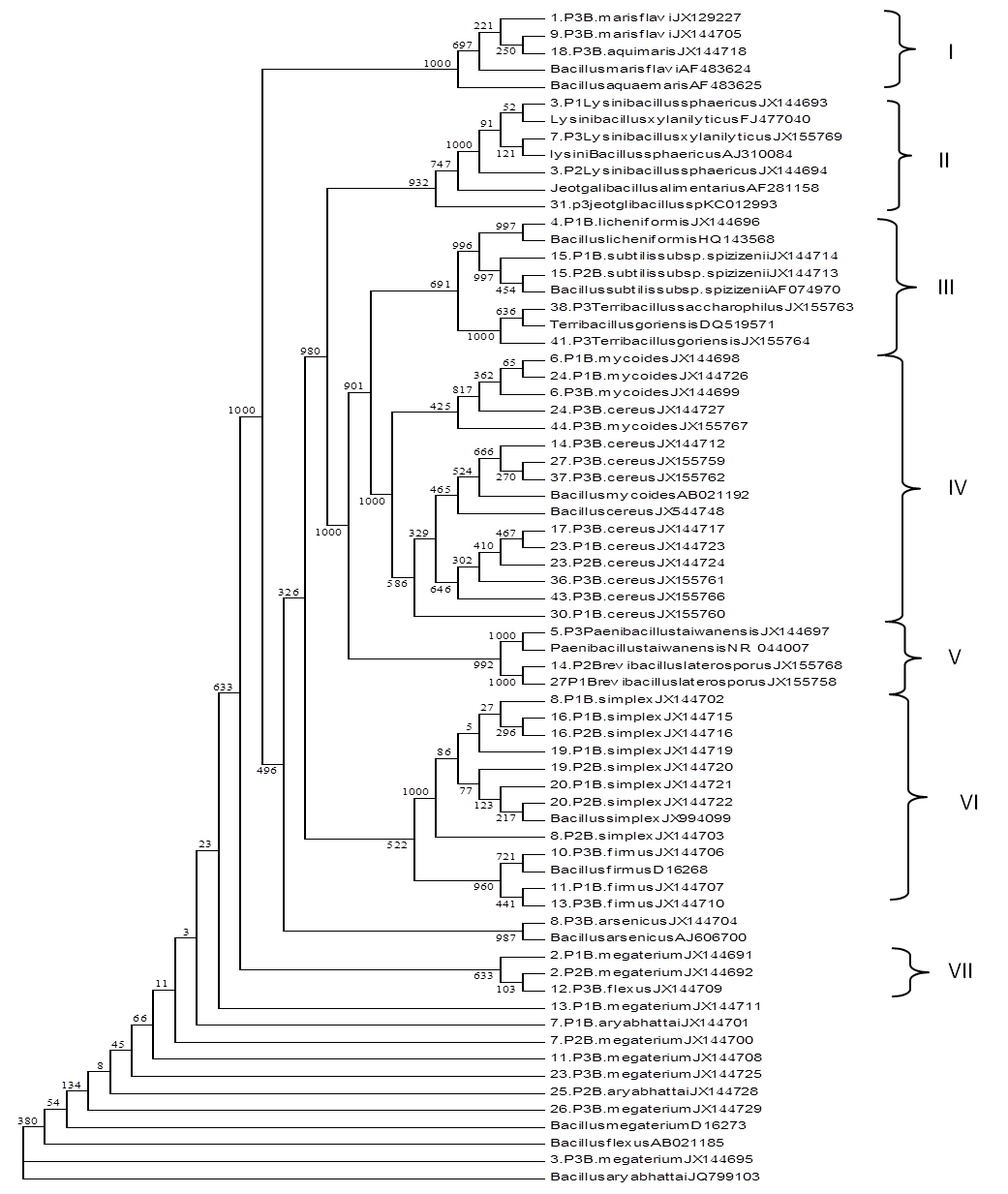


Fig 3(b)

Supplement: Supplementary file 3 — Authors’ original file for figure 3 [file 40064_2013_657_MOESM3_ESM.docx]
